# Supplementation of High Velocity Nasal Insufflation with a Nonrebreather Mask for Severe Hypoxemic Respiratory Failure in Adult Patients with COVID-19

**DOI:** 10.1155/2022/5004108

**Published:** 2022-05-24

**Authors:** Jessica S. Whittle, Jigme Sethi, Leonithas I. Volakis, Jeremy Greenberg

**Affiliations:** ^1^Vice Chair of Research, Dept. of Emergency Medicine, University of Tennessee, Chattanooga/ Erlanger Health, Chattanooga, TN, USA; ^2^VP of Clinical Research, Vapotherm Inc., Exeter, NH, USA; ^3^Dept. of Respiratory, Critical Care, and Sleep Medicine, University of Tennessee, Chattanooga/ Erlanger Health, Chattanooga, TN, USA; ^4^Research Scientist, Vapotherm Inc., Exeter, NH, USA

## Abstract

The unique clinical features of COVID-19-related acute hypoxemic respiratory failure, as well as the widespread impact leading to resource strain, have led to reconsiderations of classic approaches to respiratory support. HFNO includes high flow nasal cannula (HFNC) and high velocity nasal insufflation (HVNI). There are currently no widely accepted criteria for HFNO failure. We report a series of three patients who experienced COVID-19-related acute severe hypoxemic respiratory failure. Each patient was initially managed with HVNI and had a ROX index < 3.85, suggesting HFNO failure was likely. They were subsequently managed with a nonrebreather mask (NRM) overlying and in combination with HVNI at maximal settings and were able to be managed without the need for invasive mechanical ventilation.

## 1. Introduction

The unique clinical features of COVID-19-related acute hypoxemic respiratory failure, as well as the widespread impact leading to resource strain, have led to reconsiderations of classic approaches to respiratory support. During the early phase of the COVID-19 pandemic, high flow nasal oxygen (HFNO) was seldom used due to concerns of aerosol production and possible harm from delayed intubation. The technology has subsequently been shown to be safe [1–3], and there is emerging evidence suggesting use of HFNO is associated with decreased mortality [4], reduced need for intubation, increase in ventilator-free days, and reduced ICU length of stay [5–10]. HFNO is currently recommended for acute hypoxemic respiratory failure not responsive to low-flow oxygen by the Society of Critical Care Medicine, Surviving Sepsis Campaign, and European Society of Intensive Medicine [11].

HFNO includes high flow nasal cannula (HFNC) and high velocity nasal insufflation (HVNI). HFNO systems provide oxygen-rich, heated, humidified gas to the patient's nose at supraphysiologic flows sufficient to deliver a constant, precisely set high FiO_2_ [12, 13]. HFNC flow rates reach up to 60 L/min, whereas HVNI delivers similar quantities of oxygen at flow rates up to 40 L/min due to increased velocity derived from a lower flow with a higher level of kinetic energy in the delivered gas. Both technologies facilitate rapid deadspace flush [14] Exhalation is to the open air in all HFNO therapies. Mechanistically, HFNO provides high FiO2, reduces dead space, provides low levels of PEEP [15], and decreases breathing frequency and work of breathing [13].

There are currently no widely accepted criteria for HFNO failure. However, patients who require vasopressor support [16, 17] and whose respiratory rate and thoracoabdominal asynchrony are not rapidly relieved with HFNO are potentially at high risk of HFNO failure [18]. The “ROX Index” was developed to aid in the prediction of clinical outcomes of hypoxemic patients treated with HFNO. It is calculated as the ratio of SpO_2_/FiO_2_ to respiratory rate (RR). A ROX Index > 4.88, measured at 12 hours after treatment initiation, suggests the patient is unlikely to progress to mechanical ventilation (hazard ratio: 0.273, *p* = 0.002) [16, 19]. A ROX index < 3.85 suggests HFNO failure is likely and intubating the patient should be strongly considered [19]. The ROX index has been subsequently utilized and found to correlate with need for mechanical ventilation in patients with COVID-19 [20, 21].

We report a series of three patients who experienced COVID-19-related acute severe hypoxemic respiratory failure. Each patient was initially managed with HVNI (40 L/min max) and had a ROX Index < 3.85, suggesting HFNO failure was likely. They were subsequently managed with a nonrebreather mask (NRM) overlying and in combination with HVNI at maximal settings and were able to be managed without the need for invasive mechanical ventilation. In an effort to avoid respiratory suppression, sedating medications were avoided with the exception of minimal narcotic pain control provided as needed. No written consent has been obtained from the patients as there is no patient identifiable data included in this case report.

### 1.1. Patient 1

A 57-year-old female with a past medical history of diabetes and hypertension presented to the Emergency Department (ED) complaining of abdominal pain and diarrhea for 7 days, with cough, fever, and myalgia. Initial vital signs were temperature of 39.1°C, heart rate of 104 beats per minute (bpm), blood pressure of 143/64 mmHg, respiratory rate of 17 breaths per minute, and oxygen saturation of 89% on room air. She appeared nontoxic and was speaking in full sentences. Laboratory evaluation was unremarkable, and nasopharyngeal PCR testing for SARS-CoV-2 virus was positive. Chest X-ray showed patchy airspace disease.

Her hypoxemia was initially managed with supplemental oxygen at 3 L/min via nasal cannula (NC). On hospital day 2, she had a rapid clinical decline. HVNI and self-proning were initiated due to O_2_ sat (78%) on 6 L NC. After HVNI, her respiratory rate subsequently decreased to 28 breaths per minute and her O_2_ sat improved to 90%.

By day 5, the patient's hypoxemia had progressively worsened despite maximal settings until her O_2_ sat was 83% (ROX Index = (83/1)/33 = 2.51). The patient expressed she did not want intubation unless as the “last resort” though acknowledged feeling “exhausted” from high work of breathing. An attempt was made to honor her wishes by placing a NRM at 15 L/min over the HVNI cannula. Her oxygen saturation improved to 92%, and her respiratory rate decreased to 25 breaths per minute. This therapy stabilized the patient and continued for 3 more days followed by progressive deescalation until she was discharged home on day 16 without supplemental oxygen.

### 1.2. Patient 2

A 42-year-old male with no medical history presented to the ED with 5 days of dyspnea with fevers, chills, and nonproductive cough. Vital signs were temperature of 38.1°C, heart rate of 116 bpm, blood pressure of 100/61 mmHg, respiratory rate of 32 breaths per minute, and oxygen saturation of 47% on room air. He appeared ill and distressed. Despite critical hypoxemia, he was able to speak in truncated sentences, and his lungs were clear to auscultation. The remainder of his exam was unremarkable.

Laboratory evaluation revealed leukocytosis and elevated C-reactive protein, D-dimer, and aminotransferases. An ABG showed pH 7.38, PaCO_2_ 29, and PaO_2_ 31. Chest X-ray showed bilateral, perihilar, and basilar airspace disease, and PCR testing was positive for SARS-CoV-2. Treatment with HVNI was initiated at 40 L/min and FiO_2_ 90%. His oxygen saturation improved to 90% (ROX HVNI = (90/.9)/32 = 3.13).

On day 3, his oxygen saturation decreased to 84% despite FiO_2_ of 100%. Clinically, he was in extremis. Nonetheless, the patient requested to delay mechanical ventilation so that he could continue video chats with his family. To honor the patient's wishes, additional oxygen was administered from a NRM at 15 L/min placed over the HVNI cannula. His oxygen saturation improved to 93%, and he reported subjective improvement in his respiratory effort.

On day 5, the patient improved and was weaned to 30 L/min and FiO_2_ of 70%. Then on day 7, he again deteriorated and HVNI was escalated back to maximal support. Computed tomographic (CT) pulmonary angiography of the chest revealed multifocal, bilateral, ground-glass opacities, and dense consolidation, as well as multiple segmental and subsegmental pulmonary emboli. Despite HVNI, his oxygen saturation was 87%. To again avoid mechanical ventilation, a NRM was successfully used in conjunction with NVNI. Therapy continued, and the patient improved until day 17, when HVNI was discontinued, and supplemental oxygen was given through a nasal cannula at 10 L/min. His ICU course was then complicated by life-threatening epistaxis and delirium. The patient required an additional 10 days of hospitalization until he was discharged home on day 36 without the need for supplemental oxygen.

### 1.3. Patient 3

A 37-year-old female gravida 6 para 5 at 40 weeks estimated gestational age with no medical history presented to the obstetrics triage unit with contractions. Two weeks prior, she asymptomatically tested positive for the SARS-CoV-2 virus on routine testing. She had no viral symptoms but was admitted due to signs and symptoms concerning for acute fatty liver of pregnancy and preeclampsia. Due to fetal distress, she underwent emergent cesarean section and was extubated without difficulty. Her immediate postoperative course was uncomplicated. Her liver function tests and coagulation profile improved following delivery.

On hospital day 2, she developed mild hypoxemia and radiographic multifocal airspace disease. By day 4, she developed rapidly progressive respiratory failure with hypoxemia. Her oxygen saturation on 9 L/min was 81%, and respiratory rate was 43 breaths per minute. She was then escalated to HVNI at 40 L/min and FiO_2_ of 100%. Her oxygen saturation improved to 88%, and her respiratory rate decreased to 36 breaths per minute (ROX HVNI = (88/1)/36 = 2.44).

A trial of additional supplemental oxygen through a NRM at 15 L/min placed over HVNI was performed, and her oxygen saturation improved to 94%. Her respiratory rate decreased to 32 breaths per minute. She steadily improved, and respiratory support was slowly weaned until she was discharged home on hospital day 11 without need for supplemental oxygen.

## 2. Discussion

COVID-19 has intermittently caused a worldwide resource-challenged health systems, and HFNO has become a widespread recommended therapy for select patients [22]. Recommendations for management of COVID-19-related acute severe hypoxemic respiratory failure have some agreement in that low-flow oxygen therapy should be utilized first, followed by HFNO or NIPPV (if HFNO is not first available), and then to consider intubation with a lung-protective ventilation strategy of low tidal volume with sufficient PEEP for lung recruitment [2] [23]. However, patients that refuse intubation or situations in which ventilators become limited represent a more challenging scenario.

This report provides a series of cases, wherein COVID-19 patients with severe acute hypoxemic respiratory failure were clinically stabilized with a NRM utilized in conjunction with HVNI. This dual-modality treatment option provided positive patient clinical outcomes without the need for intubation, while simultaneously respecting patient autonomy. On a broader note, this represents a simple, relevant, and successful treatment option for patients with severe respiratory failure that should be further researched. Based on the perceived positive outcome for these three patients, this approach has become routinely adopted in our institution for patients with COVID-19-related acute severe hypoxemic respiration failure.

Nasal cannula interface with the nostril, ideally, was with an open architecture. Clinicians are generally instructed to allow for at least half of the area of the nare to remain unoccluded by the nasal prong of the HVNI cannula interface. This opening permits a free flow of exhaled gas from the patient, important to facilitate flush of the accessible extra-thoracic nasal/pharyngeal deadspace. However, this open aperture also provides an opportunity for entrainment of room air (Bernoulli effect). This issue is not typically clinically significant. Groves and Tobin measured pressures with volunteers using high flow nasal cannulas and open and closed mouths [15]. The high flow of air into the small nasal chamber created measured elevations of nasal pressure linearly increasing with flow rate, which act to prevent entrainment of room air and create positive end expiratory pressure. However, the pressure rise was markedly attenuated when the mouth was open—as is true for many highly tachypneic patients—and this is the scenario in which entrainment of NRM 100% oxygen from the mouth and even the nose can be beneficial. The safety of such a configuration is not likely impacted, as the primary gas source for the patient is provided through the HVNI, which should be set to meet patient needs. The incorporation of a NRM would not negatively impact the washout effect of the high flow oxygen therapy, as the gas available at the mouth and nose for entrainment will be oxygen rich, and the mask is designed to flush exhaled gas as part of normal operation.

There has been much discussion about the possibility of worse outcomes for patients for whom mechanical intubation is delayed, largely based on two papers. The first, in 2015 by Kang et al., reports dramatically different mortality rates comparing patients who were intubated early in the course of critical illness versus late [24]. These findings were discussed at length in a review by Ricard, suggesting that confounding variables likely played a role in why the mortality rates reported in this paper seem more extreme than those reported in other studies [25, 26]. The second study by Miller et al. was a retrospective database analysis reporting that, amongst patients who failed primary therapy and later required mechanical ventilation, patients who had been primarily treated with HFNC had higher mortality than those treated with BiPaP [27]. Unfortunately, the sample sizes are extremely different (2,241 HFNC vs. 32,761 BiPaP), and the initial indications for treatment were not available for a majority of the patients in the HFNC group. These issues suggest that the practice pattern in this hospital system is extraordinarily biased towards the use of BiPaP. It is unknown whether the bias is a result of physician comfort, scientific opinion, supplies available, or patient population. It is impossible to know from the data reported whether the two patient groups were similar enough for comparison and it is important to note that the trend to higher mortality for treatment failures was not observed in all subgroups (i.e., patients with pneumonia). Nonetheless, these reports provide valuable indicators that the proper management of patients requiring respiratory is highly nuanced and much remains unclear.

In addition to a lack of benefit being identified from early intubation [28], a recent meta-analysis of 12 studies including 8944 critically ill patients with COVID-19 showed that there was no morbidity or mortality difference in and early vs. late intubation management strategy, thus concluding that a wait and see approach may yield fewer intubations [29].

Data from multiple regions and populations throughout the world have shown considerable variation in practice regarding the timing and strategy for mechanical ventilation of patients with COVID-19-related acute respiratory failure. Outcomes remain highly variable and likely multifactorial and an optimal strategy has yet to be fully elucidated [30–34]. For patients who decline intubation and mechanical ventilation, yet for whom aggressive care is not yet futile, utilization of a NRM with HVNI may be a consideration.

## 3. Conclusion

Patients with persistently high oxygen requirements often have prolonged clinical courses of illness, higher morbidity, and potentially higher mortality rates. Thus, it seems that the concept described in this work may be worthy of consideration in a select group of patients, particularly those for whom intubation and mechanical ventilation is not appropriate or desired for end-of-life care or in extreme environments in which respiratory support resources are limited.

## Data Availability

All data are present in the manuscript.
